# Regulation of VEGF-A expression and VEGF-A-targeted therapy in malignant tumors

**DOI:** 10.1007/s00432-024-05714-5

**Published:** 2024-04-30

**Authors:** Yan Kang, Huiting Li, Yiping Liu, Zheng Li

**Affiliations:** 1 NHC Key Laboratory of Carcinogenesis, National Clinical Research Center for Geriatric Disorders, Key Laboratory of Carcinogenesis, Chinese Ministry of Health, Department of Oncology, Xiangya Hospital, Central South University, Changsha, Hunan China; 2 Cancer Research Institute, School of Basic Medical Science, Central South University, Changsha, Hunan China

**Keywords:** VEGF-A, Angiogenesis, Expression regulation, Tumor immunity, Bevacizumab, Traditional Chinese medicine

## Abstract

Vascular endothelial growth factor A (VEGF-A), a highly conserved dimeric glycoprotein, is a key regulatory gene and a marker molecule of angiogenesis. The upregulation of VEGF-A facilitates the process of tumor vascularization, thereby fostering the initiation and progression of malignant neoplasms. Many genes can adjust the angiogenesis of tumors by changing the expression of VEGF-A. In addition, VEGF-A also exhibits immune regulatory properties, which directly or indirectly suppresses the antitumor activity of immune cells. The emergence of VEGF-A-targeted therapy alone or in rational combinations has revolutionized the treatment of various cancers. This review discusses how diverse mechanisms in various tumors regulate VEGF-A expression to promote tumor angiogenesis and the role of VEGF-A in tumor immune microenvironment. The application of drugs targeting VEGF-A in tumor therapy is also summarized including antibody molecule drugs and traditional Chinese medicine.

## Introduction

The VEGF (Vascular endothelial growth factor) family members are involved in various biological processes, such as angiogenesis, lymphopoiesis, inflammation, oxidative stress, lipid metabolism, and so on (Zhou et al. 2021a, b). VEGF family includes the following members: VEGF-A, VEGFB, VEGFC, VEGFD, placental growth factor (PlGF), and EG-VEGF (Ferrara 2011). The human VEGF-A gene locus on chromosome 6p21.1 includes eight exons and seven introns. After alternative splicing of VEGF-A mRNA, VEGF165, VEGF121, VEGF206, and other isoforms with different lengths appear. Exons 1–5 and 8 are the same region in all subtypes. However, differences in exons 6–7 lead to changes in affinity with heparin and heparin sulfate proteoglycans, which change the ability of VEGF-A to bind different receptors and extracellular matrix components, and then lead to different subtypes with various biological characteristics (Braile et al. 2020; Wiszniak and Schwarz 2021).

VEGF-A binds vascular endothelial growth factor receptor VEGFR1 or VEGFR2 and then activates downstream signals to promote angiogenesis (Grassot et al. 2006). VEGFRs possess an extracellular ligand-binding domain consisting of seven immunoglobulin-like loops along with a transmembrane domain, a juxtamembrane domain, a split TK domain, and a C-terminal tail. They have different ligand-binding properties and biological functions (De Falco 2012; Jeltsch et al. 2013). In response to ligand binding, VEGFR1 and VEGFR2 dimerize to form homodimers or heterodimers (White and Bix 2023). VEGFR2 is the main binding receptor of VEGF-A and the key regulator for angiogenesis. VEGF-A binds to the extracellular domain of VEGFR2, leading to the autophosphorylation of its tyrosine residues and subsequent activation of downstream signaling pathways that facilitate endothelial cell proliferation (Wang et al. 2021a, b; Watari et al. 2020). VEGFR1 competes with VEGFR2 for VEGF-A binding due to its high affinity, but it exhibits weak autophosphorylation in the tyrosine kinase domain with limited regulatory ability for angiogenesis (Zhou et al. 2021a, b; Wiszniak and Schwarz 2021). Koizumi K et al found that VEGF165, a subtype of VEGF-A, activates VEGFR1 and regulates the motility of melanoma cells through signals mediated by PI3K/AKT kinase pathway (Koizumi et al. 2022).

In 1971, Judah Folkman proposed that tumor growth depended on angiogenesis, and tumor lesions might be alleviated by starvation by cutting off the blood supply (Folkman 1971). VEGF-A was isolated and identified in 1989 (Leung et al. 1989). In 1993, Kim and colleagues discovered monoclonal antibodies that could target and neutralize VEGFAs and inhibit tumor growth in preclinical studies (Kim et al. 1993). Recombinant humanized VEGF-A-specific monoclonal antibody, bevacizumab, was approved by the U.S. Food and Drug Administration (FDA) in 2004 for the first-line treatment of metastatic colorectal cancer (Hurwitz et al. 2004). VEGF-A has been recognized as a significant pro-angiogenic factor, with its expression alterations and structural modifications being implicated in cancer progression and associated with patients’ overall survival and treatment response (Elebiyo et al. 2022; Al Kawas et al. 2022). This review primarily concentrates on the upstream mechanisms governing VEGF-A expression in tumors, the involvement of VEGF-A in tumor immunity, as well as the investigation of clinical drugs and traditional Chinese medicine resources targeting VEGF-A.

## Regulation of VEGF-A expression in tumors

The regulation and control of transcription is an indispensable link in the process of gene expression. HIF1α-VEGF-A pathway is the strongest and most well researched signal pathways to promote angiogenesis. With the continuous growth of tumors, insufficient blood supply leads to hypoxia and necrosis of cells, which in turn induces HIF1α overexpression. HIF1α directly promotes the transcription of VEGF-A, which combines with VEGFR2 to further induce neovascularization (Liu et al. 2022a, b; Chen et al. 2023). Mielcarska S et al found that the expression of HIF1α and VEGF-A were considerably increased in tumor-free marginal tissues, revealing that non-cancerous cells in the tumor microenvironment may be an important source of VEGF-A expression (Mielcarska et al. 2021). Multiple genes regulate VEGF-A expression and function via HIF1α/VEGF-A signaling pathway. In lung adenocarcinoma cells, high expression of progesterone membrane receptor α (MPRα) increases HIF1α-induced secretion of VEGF-A into the media, which promotes the migration and tube formation of human umbilical vein endothelial cells (HUVECs) (Qin et al. 2020; Xia et al. 2022). Deacetyltransferase HDAC1 is able to inhibit HIF1α ubiquitination degradation process, which in turn activates HIF1α/VEGF-A signaling pathway to promote angiogenesis in colorectal cancer (Chen et al. 2020).

Except HIF1α, other molecules have the ability to regulate the expression of VEGF-A by transcription regulation. Transcription 3 (STAT3) is also a direct upstream factor of VEGF-A, which binds to VEGF-A promoter and stimulates its transcription. Tribbles pseudokinase 3 (TRIB3) can cooperate with STAT3 to increase VEGF-A expression and promote colorectal cancer angiogenesis (Chen et al. 2022). Leucine-rich α-2-glycoprotein 1 (LRG1) activates VEGF-A expression through the SRC/STAT3 pathway and promotes angiogenesis in gastric cancer (He et al. 2022). RNA-binding motif 4 (RBM4) enhances the expression of VEGF-A via NF-κB signaling pathway and then promotes hepatocellular carcinoma progression (Han et al. 2023). REC8 inhibits angiogenesis and tumor progression by decreasing VEGF-A expression in the gastric cancer. Mechanically, the loss of REC8 expression significantly promotes NF-κB binding to the VEGF-A promoter, which in turn enhances VEGF-A transcription (Liu et al. 2020). C-terminal tensin-like (CTEN) decreases VEGF-A expression via down regulation β-catenin, which in turn inhibits angiogenesis and cancer cell growth in breast cancer (Lu et al. 2021). In pan-cancer, VEGF-A mRNA tissue expression is significantly increased in TP53-mutant adenocarcinoma (but not squamous carcinoma) compared to TP53 wild-type tumors (Li et al. 2020a, b).

N6-methyladenosine (m6A) is the most prevalent internal modification of mRNA which has an effect on RNA stability, translation, and secondary structure (Boulias and Greer 2023). M6A modification of VEGF-A is also upregulation of VEGF-A in various tumors. Mechanistically, METTL3 enhances m6A modification of VEGF-A which improves it interaction with IGF2BP2/3 and impedes the degradation of VEGF-A mRNA (Liu et al. 2022a, b; Yang et al. 2020). The study of Zhang G et al also showed that METTL3 could enhance VEGF-A mRNA stability and protein level in colorectal cancer (Zhang et al. 2022). In addition, m6A methylation of the internal ribosome entry site sequence A (IRES-A) at VEGF-A 5'UTR recruits the YTHDC2/eIF4GI complex to facilitate its’ cap-independent translation initiation (Zhang et al. 2023). These studies greatly enrich the research of m6A modification influencing on angiogenesis of malignant tumor, and strengthen the theoretical basis of VEGF-A target through m6A regulation on tumor treatment.

It has been shown that microRNAs regulate gene expression on a transcriptional and posttranscriptional level and play a role in a wide range of physiological and pathological processes (Sufianov et al. 2023). MiR-503-5p, miR-16-5p, miR-486, and miR-199a directly target VEGF-A to inhibit angiogenesis in colorectal cancer (Wei et al. 2022; Wu et al. 2020; Situ et al. 2022). MiR-612, miR-637, miR-874, and miR-34a-5p inhibit the development and progression of hepatocellular carcinoma by targeting VEGF-A (Castanhole-Nunes et al. 2022; Niu et al. 2022). MicroRNAs, such as miR-4316 and miR-205-5p, inhibit gastric cancer cells reproduce and migrate by targeting VEGF-A (Mousa et al. 2020; Zhang et al. 2021). MiR-574-5p targets tyrosine–protein phosphatase non-receptor type 3 (PTPN3) to enhance phosphorylation of ERK and VEGF-A expression, thereby promoting angiogenesis in gastric cancer (Zhang, 2020a, b, c, d).

On the other hand, long non-coding RNA (lncRNA) and circular RNA (circRNA) regulate the expression of VEGF-A to affect tumor angiogenesis through competitive endogenous RNA (ceRNA) mechanism. LncRNA TUG1, as a ceRNA sponging miR-29c-3p to up-regulate VEGF-A expression, promotes stomach adenocarcinoma angiogenesis (Jin et al. 2021). LncRNA-UCA1 interacts with miR-383, which in turn enhances VEGFA's expression in lung adenocarcinoma (Tang et al. 2023). LUCAT1 promotes VEGF-A expression in lung cancer by regulating the miR-4316 as an endogenous competitive RNA (Wang et al. 2022). Circ-0056618 in colorectal cancer affects cell proliferation, migration, and angiogenesis through the sponging effect of miR-206 (Zheng et al. 2020). VEGF-A is regulated by CircCCT3/miR-613 and CircUBAP2/ miR-199a to enhance colorectal cancer angiogenesis (Li et al. 2020a, b; Dai et al. 2020). Circ-0001178/miR-382 and NORAD/miR-211-5p regulate hepatocellular carcinoma angiogenesis via VEGF-A (Sun et al. 2021; Gao et al. 2020). In brain glioma, LINC01116 regulates the expression of VEGF-A by competitively absorbing miR-31-5p (Ye et al. 2020). Circ-RPL15 up-regulates VEGF-A by competitively binding miR-146b-3p, thereby triggering proliferation and migration potential of glioma (Wang et al. 2020a, b). Except as ceRNA, LncRNA RP11-732M18.3 promotes EP300 into the nucleus to enhance transcription and secretion of VEGF-A, and thus promote angiogenesis in glioma (Kang et al. 2022). TPT1-AS1 regulates angiogenesis in colon cancer by regulating expression and stability of VEGF-A mRNA through NF90 (Zhang et al. 2020a, b, c, d).

**Fig. 1:**
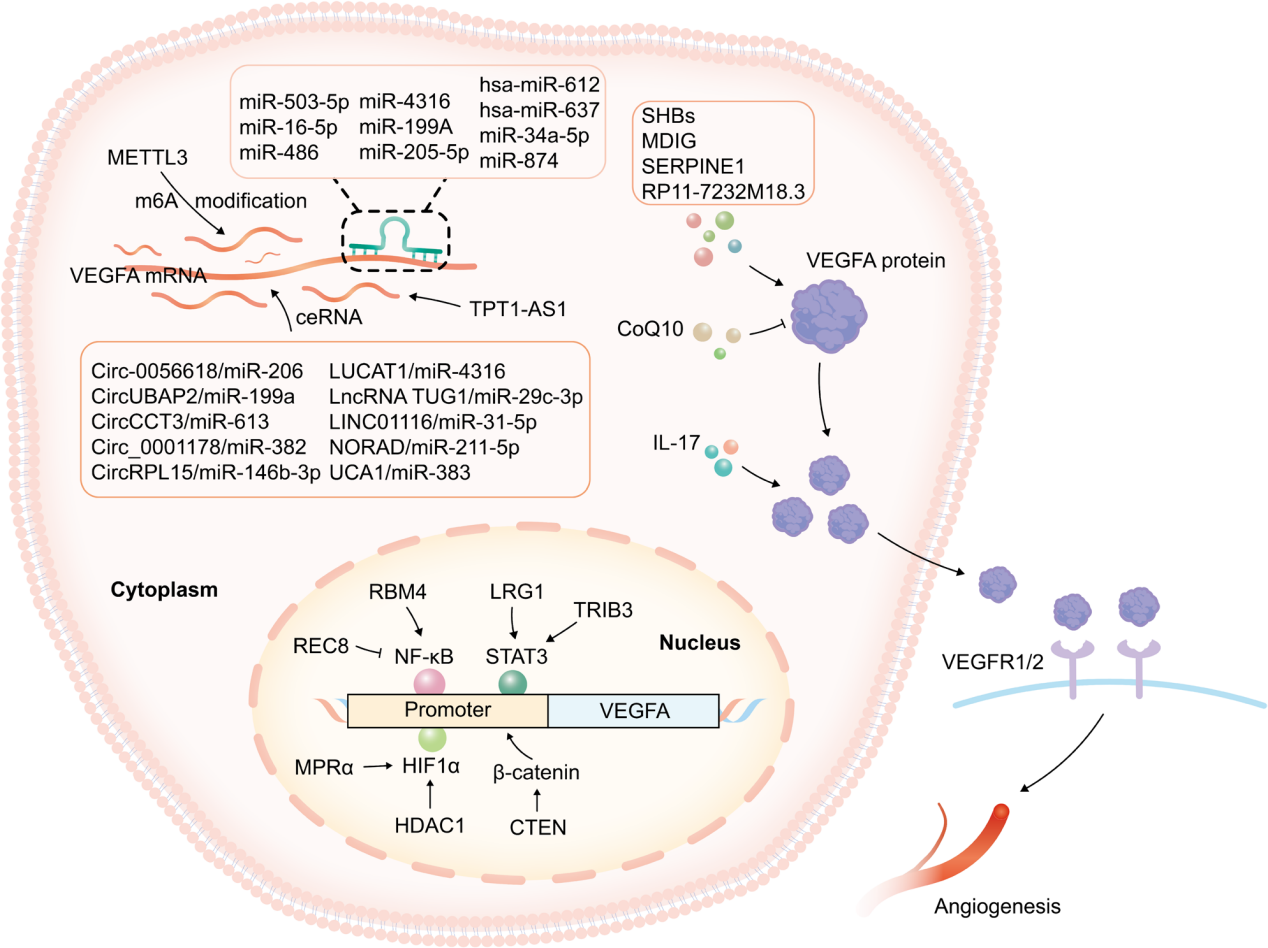
Molecular mechanism of VEGFA level regulation in various tumors

The protein expression of VEGF-A is also affected by several molecules. SERPINE1 promotes the proliferation of breast cancer cells by regulating the protein level of VEGFA (Zhang et al. 2020a, b, c, d). CoQ10 inhibits the protein expression of VEGFA/VEGFR2 and then prevents the progression of breast cancer (Abdi et al. 2020). Qian XL et al showed that IL-17 could increase tumor VEGFA secretion to stimulate angiogenesis, and then promoted triple-negative breast cancer progression (Qian et al. 2020). MDIG is an oxygen-sensitive protein that promotes angiogenesis and glioma growth by activating the EGFR/p-EGFR/VEGFA/VEGFR1/R2 pathway (Zhou et al. 2021). SHB, a small protein of hepatitis B virus (HBV) surface antigen, is the most abundant protein in HBV and has a close clinical relationship with hepatocellular carcinoma. Wu SX et al demonstrated that SHBs could up-regulate VEGFA protein expression both in vivo and in vitro, thereby enhancing the angiogenesis of hepatocellular cancer cells (Wu et al. 2022). Molecular mechanisms of VEGFA level regulation in various tumors are summarized in Figure 1.

## VEGFA and tumor immunity

**Fig. 2:**
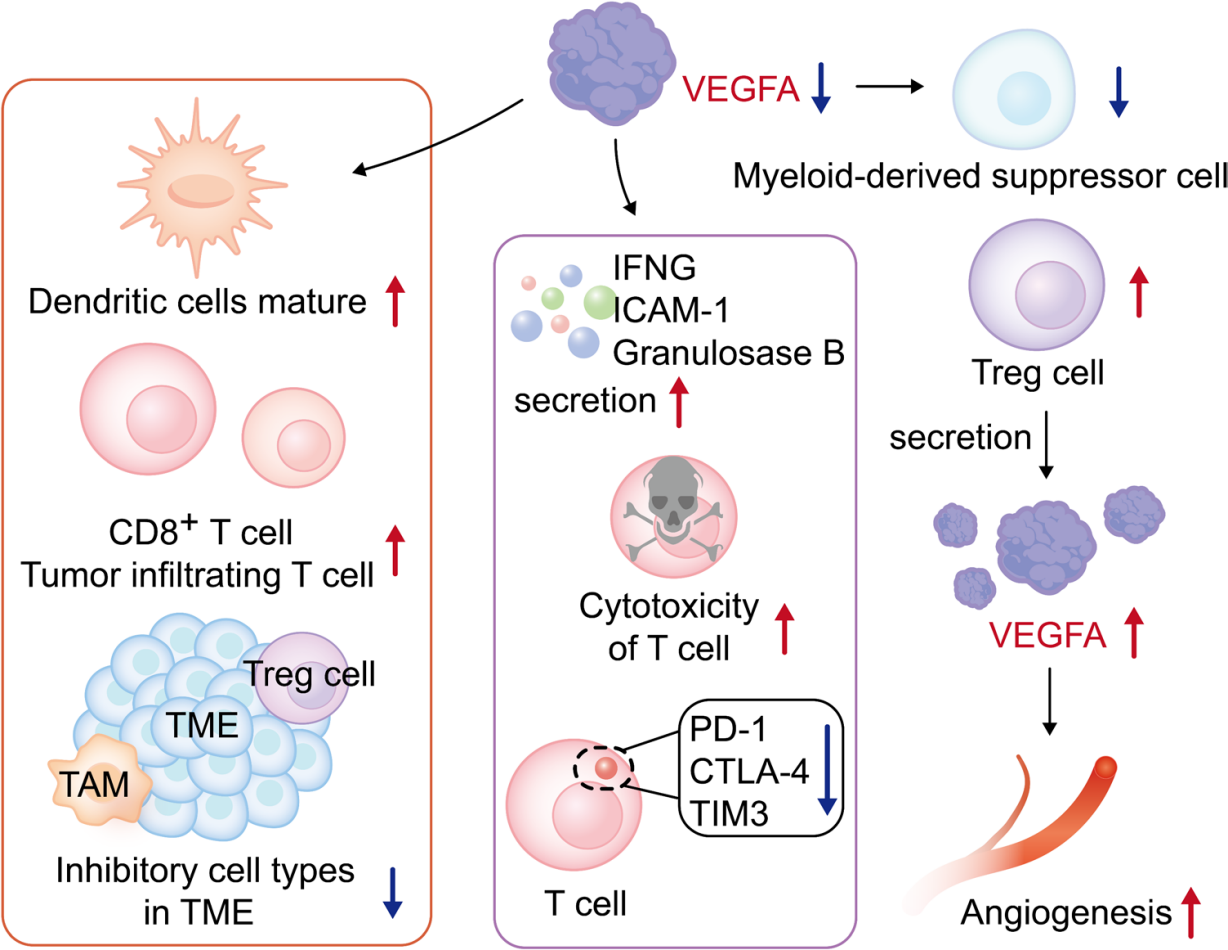
VEGFA and tumor immunity

In addition to being an important angiogenic factor, VEGF also modulates the microenvironment of tumors (Fukumura et al. 2018). Increased VEGFA expression can lead to immunosuppression by inhibiting dendritic cell (DC) maturation, reducing T cell tumor infiltration, and promoting suppressive cell types in the tumor microenvironment (Ribatti 2022). VEGF inhibits the differentiation of monocytes into DC, and bevacizumab and sorafenib can reverse the inhibitory effect of recombinant VEGF on DC differentiation (Alfaro et al. 2009). Yulia Nefedova et al demonstrated that VEGF-A promotes circulating myeloid-derived suppressor cell (MDSC) accumulation by activating the JAK2-STAT3 pathway (Nefedova et al. 2004). VEGFA secreted from colorectal carcinoma cells activated CXCL1 production in tumor-associated macrophages, which recruited CXCR2-positive MDSCs to form a pre-metastatic niche that ultimately promoted liver metastases (Wang et al. 2017). VEGF-driven angiogenesis forms a barrier to T cell infiltration by decreasing endothelial intercellular adhesion molecule 1 (ICAM-1) expression (Griffioen et al. 1996). Abnormal structure and function of the tumor vasculature form a barrier to CD8^+^ T cell infiltration (Schaaf et al. 2018). In ovarian cancer patients, ascites-derived T cells secrete VEGFA and express VEGFR2 in response to activation. VEGFA-VEGFR2 axis directly inhibits T cell proliferation and function (Gavalas et al. 2012). VEGFA can limit T cell recruitment into tumors and promote T cell exhaustion (Li et al. 2023). Meanwhile, regulatory T cells (Tregs) can secrete a large amount of VEGFA which induces endothelial cell neovascularization (Kajal et al. 2021). In hepatocellular carcinoma, increased VEGFA expression promotes the expression of PD-1, CTLA-4 and Tim-3 on T cells, as well as inhibits the secretion of interferon γ (IFNG) and granulosase B (Deng et al. 2020). In breast cancer, the VEGFA expression is positivity significantly correlated with PD-L1, and VEGFA may be a predictor of immune characteristics and serves as a useful biomarker for immune targeted therapy (Fujii et al. 2020). Other studies have found that the CXC chemokine-VEGFA network is significantly correlated with immune cell infiltration in colon cancer (Castanhole-Nunes et al. 2022; de Almeida et al. 2020). The interaction of VEGFA and tumor immunity are summarized Figure 2.

## Drugs targeting VEGFA in cancer therapy

Monoclonal antibody therapy targeting VEGFA is a commonly used strategy in tumor treatment (Estrada et al. 2019). The monoclonal antibody bevacizumab is a recombinant human monoclonal G1 immunoglobulin that mainly targets tumor blood vessels and exerts anti-tumor effects. It specifically binds to VEGFA, blocks its binding to the receptor VEGFR, and then inhibits the signal transduction (Estrada et al. 2019; Nakai and Matsumura 2022; Clou and Luque 2022). Bevacizumab marks the beginning of a new series of anti-cancer therapies and remains the most widely characterized anti-angiogenic therapy. Initially, it was approved for treating metastatic colorectal cancer with chemotherapy resistance. Currently, metastatic breast cancer, non-small cell lung cancer, glioblastoma, and other cancers are also being treated with bevacizumab (Garcia et al. 2020). Common toxicities reported with bevacizumab include hypertension, proteinuria, and gastrointestinal perforation. Although it can improve the overall survival of tumor patients, most patients eventually develop drug resistance, resulting in a poor clinical prognosis (Rehman et al. 2022). High serum levels of VEGFA and placental growth factor PlGF may be the basis of bevacizumab resistance in patients with metastatic colorectal cancer. Regardless of baseline VEGFA and PlGF levels, patients with bevacizumab-induced resistance were found to benefit from aflibercept as a second-line treatment (Van Cutsem et al. 2020).

Small molecule inhibitors of VEGFR2 were first reported in 1996 (Strawn et al. 1996). Sorafenib, apatinib, pazopanib, and axitinib are all receptor tyrosine kinase inhibitors (RTKIs). Sorafenib inhibits VEGFR2 auto-phosphorylation (Wilhelm et al. 2004) and has good efficacy in metastatic renal cell carcinoma (Strumberg 2005). Apatinib is an oral RTKI main targeting VEGFR2 which also used in many cancer treatment (Ferrara et al. 2003). Pazopanib monotherapy has been shown to treat renal cell carcinoma (Sternberg et al. 2010). Axitinib is more selective than sunitinib for VEGFR (Hu-Lowe et al. 2008). Protein inhibitor Ramucirumab, developed by ImClone Systems Inc, binds to VEGFR2 to block VEGF binding and inhibit angiogenesis (Krupitskaya and Wakelee 2009). Several studies have shown that bevacizumab can combine with a variety of tyrosine kinase inhibitors for better anti-tumor angiogenesis therapy efficiency. In lung cancer, bevacizumab combined with apatinib inhibits the growth of NSCLC tumor cells and prolongs a tumor-bearing mouse's median survival time (Wang et al. 2020a, b). Xie H et al found bevacizumab combined with sorafenib as a salvage treatment for metastatic colorectal cancer patients who failed to various treatments (Xie et al. 2020). At the same time, bevacizumab combined with multi-kinase inhibitors targeting VEGFRs has also been found to be equally more effective against advanced solid cancers, such as melanoma (Tamura et al. 2020). Icotinib (a tyrosine kinase inhibitor that selectively targets EGFR) combined with bevacizumab has a stronger inhibitory effect on NSCLC growth in vivo than single target drugs without additional side effects (Jiang et al. 2022). The expression of VEGFA mRNA is remarkably increased in EGFR mutated advanced NSCLC, and the clinical efficacy of bevacizumab in EGFR mutated patients is better than that in wild-type carriers. The addition of bevacizumab to the EGFR inhibitor erlotinib can prolong the progression free survival of EGFR mutated NSCLC patients by more than 6 months, and benefit more in EGFR L858R mutated patients, which provides further evidence for the targeted application of VEGFA/EGFR combination (Tanaka et al. 2020; Le et al. 2021).

In addition to the combination therapy with tyrosine kinase inhibitor drugs, anti-VEGFA therapy can be combined with chemotherapy or immunotherapy in cancer treatment. Damato A confirmed that FOLFOXIRI scheme (fluorouracil, leucovorin, oxaliplatin, and irinotecan) combined with bevacizumab was one of the first-line treatment schemes with the highest activity in metastatic colorectal cancer patients without biomolecular changes (Damato et al. 2020). Bevacizumab combined with chemotherapy therapy gets more beneficial effect in advanced ovarian cancer patients (Burger et al. 2010). Bevacizumab in combination with immunotherapy has also shown encouraging results. In patients with metastatic renal cell carcinoma, intra-tumoral antigen-specific CD8^+^ T cell migration has been found to increase when combined with bevacizumab and atezolizumab (anti-PD-L1) (Lopes-Coelho et al. 2021). Anti-PD-L1 antibody combined with bevacizumab inhibits ovarian tumor growth and increases CD8^+^ T cell infiltration compared to anti-VEGF monotherapy (Ishikura et al. 2022). In NSCLC patients with EGFR mutations and liver or brain metastases, the OS improvements were sustained in group treated with atezolizumab (PD-L1 antibody) plus bevacizumab and carboplatin (Reck et al. 2019). Sugawaras' study showed that atezolizumab targeting PD-L1 combined with bevacizumab could be used as the first-line treatment for advanced hepatocellular carcinoma (Sugawara et al. 2021). The tyrosine kinase inhibitor axitinib can be used in combination with pembrolizumab (anti-PD-1) to treat patients with advanced renal cell carcinoma (Atkins et al. 2018). The anti-PD-L1 drug avelumab is better in combination with axitinib for patients with advanced renal cell carcinoma (Motzer et al. 2019). Adoptive transfer of chimeric antigen receptor CAR-T cells has become an effective immunotherapy against some hematological malignancies. Studies have found that co-administration of anti-VEGFA antibody in vivo promotes the persistence of CAR T cells and tumor control (Lanitis et al. 2021).

Extracts from various Chinese herbs and plants have also been found to have the potential to inhibit the angiogenesis in various cancers by regulating VEGFA. Through screening a natural compound library containing 330 small molecules in the MDA-MB-231 cell line, Zou et al. found emodin, a natural anthraquinone derivative, could inhibit VEGFA expression. Seryl-tRNA synthetase (SerRS) is a potent transcriptional repressor of VEGFA. Emodin activates SerRS expression to inhibit VEGFA transcription via targeting nuclear receptor corepressor 2 (NCOR2) and releasing it from the SerRS promoter. In vivo, emodin can effectively inhibit the vascular development of zebrafish and block tumor angiogenesis in TNBC mice (Zou et al. 2020). In colorectal cancer, the inhibitory effect of emodin on VEGFA achieved through targeting long-chain acyl-CoA synthetase 4 (ACSL4) (Dai et al. 2022). In another screening experiment in TNBC cells, Isoflavone derivative 3-(4-methoxyphenyl) quinoline-4(1H)-one (MEQ) was found that increased SerRS mRNA and protein levels downregulated VEGFA production. MEQ was also found to inhibit angiogenesis in zebrafish, as well as angiogenesis in mouse TNBC allografts and xenografts models (Zhang 2020).

Asiatic acid (AA) is one of the main components of triterpenoid extract from the natural plant *Centella asiatica*. Experiments have shown that AA could significantly inhibit angiogenesis and vascular permeability through VEGFA/VEGFR2 signaling axis, and decrease the growth and metastasis of breast cancer (Tian et al. 2021). Curcumin, a polyphenolic compound found in turmeric, is the most promising anti-cancer active molecule. Quinacrine is an acridine derivative that can be used to treat malaria. Nayak D et al found that SP cells (highly enriched cells with cancer stem cells) rich in ATP-binding cassette subfamily G member 2 (ABCG2) were able to release VEGFA and induce angiogenesis by the PI3K-Akt-eNOS cascade under hypoxic conditions. Curcumin combined with quinacrine reduces this phenomenon by inhibiting ABCG2. A similar situation was observed in patient-derived primary tissue-mediated xenograft (PDX) mice model (Nayak et al. 2023). Sanhuang Tang (SHD) is a traditional Chinese medicine formula composed of ephedra, astragalus, skullcap, angelica, and pubescens. SHD reduced cell migration and tubule formation ability of HUVECs. In breast cancer xenografts mice model, SHD could inhibit AURKA and VEGFA expression and neovascularization (Xu et al. 2020). The main ingredients of the traditional Chinese medicine formula Babaodan include bezoar, antelope horn, musk, notoginseng, margarita, and snake gall bladder. Babaodan inhibits the expression of VEGFA in with concentration-dependent manner. In co-culture system of gastric cancer cells and HUVECs, cell migration and tubule formation ability of HUVECs are all decreased after Babaodan treatment (Guan et al. 2021). Alginic acid (AA) is a naturally occurring glucuronic acid that is often rich in edible brown algae. AA activates the expression of miR-506 to inhibit STAT3 expression then downregulation of VEGFA. In NSCLC xenografts mice model, oral administration of AA could significantly decrease angiogenesis in tumor tissue (Wang, Wang, et al. 2021a, b). Bakshi HA recently found that Crocin, a dietary carotenoid extracted from saffron Himalaya, could inhibit the progression of colon cancer by blocking TNF-α/NF-κB/VEGFA pathway to inhibit angiogenesis and colorectal cancer cell metastasis (Bakshi et al. 2022). Robustaflavone (RF) is a natural biflavonoid compound that inhibits VEGFA stimulated blood vessel formation in CT-26 cell-derived mice tumor models (Sim et al. 2020). The traditional Chinese medicines target VEGFA is summarized in Table 1.

**Table 1:** Traditional Chinese medicine and plant extracts targeting VEGFA/VEGFR for cancer treatment

Name	Source	Target/Mechanism	Cancer	References
Emodin	*Rheum palmatum*	NCOR2/SerRS/VEGFA; ACSL4/VEGFA/VEGFRs	BC;CRC	Zou et al. (2020); Dai et al. (2022)
MEQ	Isoflavone derivative	MTA2/SerRS/VEGFA	BC	Zhang et al. (2020)
AA	*Centella asiatica*	VEGFA/VEGFR2	BC	Tian et al. (2021)
Curcumin; Quinacrine	*Curcuma longa* Linn; Red cinchona tree or Lai's cinchona tree	ABCG2/VEGFA	BC	Nayak et al. (2023)
San Huang Decoction	Chinese herb formula	AURKA/VEGFA	BC	Xu et al. (2020)
Babao Dan	Chinese medicine formula	VEGFA/VEGFR2	GC	Guan et al. (2021)
Alginic acid	Edible brown algae	miR-506/STAT3/VEGFA	NSCLC	Wang, Wang,et al. (2021a, b)
Crocin	Himalayan crocus	TNF-α/NF-κB/VEGFA	CRC	Bakshi et al. (2022)
RF	*Rhus succedanea*; *Garcinia multiflora*; *Ochna schweinfurthiana*; *Dietes bicolor*; *Selaginella* species	VEGFA	CRC	Sim et al. (2020)

## Summary

In summary, VEGFA plays a crucial role in regulating angiogenesis and impacting the tumor microenvironment, thereby facilitating the onset and advancement of tumors. VEGFA expression is elevated in numerous malignancies and is subject to regulation by various mechanisms. Antibody agents targeting VEGFA have gained significant popularity in the treatment of tumors, demonstrating notable efficacy. Furthermore, novel therapeutic approaches focusing on VEGFA are emerging, particularly within the realm of traditional Chinese medicine. Further clinical research is necessary to ascertain the efficacy of traditional Chinese medicine in regulating VEGFA and its potential role as an adjunct therapy. In future, combination therapy involving Chinese medicine and conventional drugs may offer enhanced treatment outcomes for patients.

## Data Availability

Not applicable.
